# ARIP1 Deficiency Facilitates the Inhibition of Neuronal Ferroptosis in Cerebral Ischemia by Activin A Through SMAD3 and p38 MAPK Signaling

**DOI:** 10.1111/cns.70615

**Published:** 2025-09-18

**Authors:** Zhulin Zou, Yunhan Zhang, Chenmeng Guo, Xinyao Qie, Daqing Xie, Lerong Wang, Zhonghui Liu, Haiyan Liu

**Affiliations:** ^1^ Key Laboratory of Pathobiology Ministry of Education, Department of Anatomy, College of Basic Medical Sciences Jilin University Changchun China; ^2^ Department of Immunology, College of Basic Medical Sciences Jilin University Changchun China

**Keywords:** Activin A, ARIP1, cerebral ischemia, ferroptosis, SLC7A11/GPX4

## Abstract

**Introduction:**

Ferroptosis is an essential pathophysiological process in cerebral ischemic injury. Activin receptor‐interacting protein 1 (ARIP1) is a negative regulator of the activin signaling pathway in neurons.

**Objective:**

This study investigated whether activin A inhibits neuronal ferroptosis and the role of ARIP1 in cerebral ischemic injury.

**Methods and Results:**

In this study, activin A increased the viability of primary neurons under conditions of oxygen–glucose deprivation (OGD). Subsequent RNA‐sequencing analysis of activin A‐treated neurons identified expression of *Slc7a11* as the ferroptosis‐associated gene with significant upregulation. Next, using the CRISPR/Cas9 system, mice were generated with a heterozygous deficiency of ARIP1 (*Arip1* ^*−/+*^), and the results revealed that the expression of GPX4 was markedly elevated and SLC7A11 was reduced in OGD‐treated *Arip1* ^*−/+*^ neurons, compared with that in wild‐type (WT) neurons, which was accompanied by an increase of p‐SMAD3 and a decrease of p‐p38 MAPK levels. In addition, our data showed that activin A activated SMAD3 and inhibited p38 MAPK phosphorylation, which differentially modulated SLC7A11 and GPX4 expression, ultimately suppressing OGD‐induced ferroptosis. Notably, *Arip1* ^*−/+*^ mice showed an improvement in neurological deficits and reduced cerebral infarction in the permanent middle cerebral artery occlusion (pMCAO) model. Furthermore, we observed that activin A exerted similar protective effects against ischemic injury in *Arip1* ^*−/+*^ mice.

**Conclusion:**

These findings indicate that downregulating the expression of ARIP1 suppresses neuronal ferroptosis by modulating SLC7A11/GPX4 expression via SMAD3 and p38 MAPK signaling, ultimately enhancing the neuroprotective role of activin A against cerebral ischemia.

## Graphical abstract

ARIP1 deficiency potentiates the neuroprotective effect of activin A against ferroptosis, attributed to regulating SMAD3 and p38 MAPK signaling.

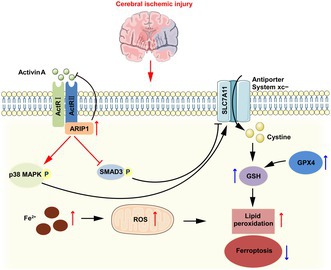

## 1 Introduction

Ischemic stroke occurs due to vascular occlusion, leading to cerebral ischemia, neuronal death, and neurological deficits [1]. While pharmacologic and mechanical thrombolysis are effective treatments, few patients achieve optimal therapeutic outcomes [2, 3, 4]. Furthermore, these interventions can trigger secondary injury, such as glutamate excitotoxicity and free radical‐induced injury, thereby aggravating the initial ischemic insult [5, 6]. Thus, alternative treatments, especially those that can prevent neuronal death, are still urgently needed.

The concept of neuroprotection emphasizes the prompt initiation of therapy following the onset of stroke to minimize neuronal death [7]. Notably, ferroptosis, an iron‐dependent form of cell death driven by the accumulation of lipid peroxidation, has been identified as an important contributor to cerebral ischemic injury [8, 9]. Our study has reported that the activity of glutathione peroxidase 4 (GPX4), a key regulator of ferroptosis, was significantly reduced after cerebral ischemia [10]. Additionally, recent research has indicated that inhibitors of ferroptosis, such as ferrostatin‐1 (Fer‐1), can mitigate neuronal damage induced by cerebral ischemia [11]. Furthermore, the expression of the *Slc7a11* gene, encoding the cystine/glutamate xCT transporter, an upstream regulator of GPX4, is increased during ferroptosis after ischemic stroke [12, 13]. Consequently, there is an urgent need to develop neuroprotective strategies targeting ferroptosis after ischemic stroke.

Activin A (Act A) is an endogenous factor with a neuroprotective role in ischemic stroke, neuropsychiatric diseases, and neurodegenerative disorders [14, 15, 16, 17]. Recent studies have demonstrated its marked protective effect against ischemia‐induced autophagy and apoptosis, highlighting its therapeutic potential in ischemic stroke [16, 18]. The intracellular signal transduction mediated by activin A mainly depends on activin A/SMADs signaling. It was also reported that Act A exerts its neuroprotective effects through the Act A/SMADs pathway after cerebral ischemia [19, 20]. Meanwhile, activin receptor‐interacting protein 1 (ARIP1) functions as a negative regulator of the Act A signaling pathway in neurons via its interaction with the ActRII receptor and SMAD3 [21, 22]. Recent research has shown that the activation of non‐SMAD‐dependent pathways can result in alterations to cell biological activities [23]. Non‐Smad dependent pathways include mitogen‐activated protein kinase (MAPKs), PI3K/AKT/mTOR, and small G protein [24]. Increasing evidence suggests that Act A, in addition to activating the Act A/SMADs signaling pathway, simultaneously inhibits the JNK and p38 MAPK pathways, thereby reducing neuronal death after cerebral ischemia [18, 25]. However, the role and mechanism of Act A and ARIP1 in neuronal ferroptosis in cerebral ischemic injury remain unclear.

In this study, we assessed the neuroprotective roles of Act A in neuronal ferroptosis following cerebral ischemia employing both in vivo permanent middle cerebral artery occlusion (pMCAO) and in vitro oxygen–glucose deprivation (OGD) models with a heterozygous deficiency of ARIP1 (*Arip1* ^*−/+*^) mice. Our data suggested that the neuroprotective role of Act A is associated with the inhibition of neuronal ferroptosis. This effect is exerted via the targeting of the SMAD3 and MAPK pathways in *Arip1* ^*−/+*^ mice, which subsequently modulates the SLC7A11/GPX4 axis. These findings provide insights into the mechanistic relationship between cerebral ischemia and ferroptosis, offering potential approaches for preventive strategies and therapeutic interventions in cerebral ischemia.

## 2 Materials and Methods

### 2.1 Animals

Eight‐week‐old C57BL/6J mice (obtained from the Beijing Vital River Laboratory Animal Technology Co. Ltd) and 8‐week‐old heterozygote *Arip1* knockout (*Arip1* ^−/+^) mice (provided by Shanghai Model Organisms Center Inc.) with the same genetic background were used for in vivo experiments. All animals were housed under controlled conditions (temperature: 21°C ± 2°C, relative humidity: 60% ± 5%, 12‐h/12‐h light/dark cycle) and had free access to food and water. All experiments followed NIH guidelines and were approved by the Experimental Animal Ethics Committee of the Basic Medical College of Jilin University.

### 2.2 The Generation of *Arip1* ^−/+^ Mice

The heterozygous *Arip1* ^−/+^ mice were generated using the CRISPR/Cas9 system. A highly active single guide RNA (sgRNA) targeting the *Arip1* gene of C57BL/6J mice was in vitro transcribed and microinjected with Cas9 mRNA into the oosperm of C57BL/6J mice. F0 generation offspring positive for the mutation were obtained by genotyping. *Arip1* homozygous null mice died during the embryonic period, whereas their heterozygous siblings (*Arip1* ^−/+^) survived. Genotyping was performed on DNA obtained from the tails of 8‐week‐old mice using a mouse tissue direct PCR kit (GKG205‐01, TIANGEN) as specified by the manufacturer. The PCR products were separated by 2% agarose gel electrophoresis.

### 2.3 OGD Treatments In Vitro

Primary neurons were cultured in MEM/EBSS (SH30024.01B, HyClone) and placed in a hypoxia incubator containing 5% O_2_, 5% CO_2_, and 90% N_2_ at 37°C for 4 h to induce OGD injury. The neurons were divided into five groups—Control (Ctrl), OGD, OGD‐Act A, OGD‐BIRB796, or OGD‐SRI‐011381 and Act A‐Anisomycin or Act A‐SB‐431542 (BIRB796 [HY‐10320, MCE] as p38 signaling pathway inhibitor, SRI‐011381 [HY‐100347, MCE] as SMAD signaling pathway agonist, Anisomycin [HY‐18982, MCE] as p38 signaling pathway agonist, and SB‐431542 [HY‐10431, MCE] as SMAD signaling pathway inhibitor). Neurons were untreated (Ctrl), stimulated with OGD, pretreated with Act A (25 ng/mL) or BIRB796 (10 μM) or SRI‐011381 (10 μM) for 30 min before undergoing OGD for 4 h, or treated with Act A (25 ng/mL) + SB‐431542 (5 μM) or Anisomycin (2 μM) for 4 h.

### 2.4 RNA Sequencing and Bioinformatic Analysis

RNA sequencing was performed on neurons pretreated with Act A and then subjected to OGD. Sequencing was carried out on the Illumina NovaSeq 6000 platform. mRNAs were considered differentially expressed using the criteria |log_2_(FoldChange)| > 1 and *p*‐value < 0.05. Bioinformatics analysis included the identification of differentially expressed genes, heat map generation, and Kyoto Encyclopedia of Genes and Genomes (KEGG) pathway enrichment analysis.

### 2.5 qPCR

Total mRNA was extracted from samples using TRIzol reagent (ThermoFisher Scientific) according to the manufacturer's instructions. Then, 2 μg of RNA was used to generate cDNA through SuperScript II reverse transcriptase (ThermoFisher Scientific). After an initial denaturation step of 95°C for 30 s, 30 cycles of PCR were performed. Each cycle consisted of a melting step at 95°C for 15 s and an annealing‐extension step for 45 s. The primer sequences are shown in Table S1.

### 2.6 Assessment of Intracellular Fe^2+^

Fe^2+^ levels were measured using an iron content assay kit (EIAFECL2, Thermo Fisher). Neurons (2 × 10^6^) and 100 mg of cortical tissue from the infarcted side of pMCAO mice were homogenized in precooled iron content buffer, centrifuged, and the supernatant was collected. Then, 50 μL of the sample was mixed with 50 μL of assay buffer, and the mixture was incubated for 30 min in the dark. Absorbance was recorded at 593 nm using a Tecan Austria reader (A‐5082, Tecan Trading AG, Männedorf, Switzerland) to calculate the Fe^2+^ content.

### 2.7 Determination of Malondialdehyde (MDA) Content

Lipid peroxidation levels were determined using an MDA assay kit (S0131S, Beyotime). Neurons (2 × 10^6^) or 100 mg of mouse brain tissues were homogenized with lysis buffer, centrifuged, and the supernatant was collected. The protein content of samples was measured using a BCA protein quantification kit as described by the manufacturer. Briefly, the MDA working solution was added to the supernatant, which was boiled at 100°C for 15 min, and then centrifuged. A 100‐μL volume of sample was used to measure the absorbance at 532 nm using a Tecan A‐5082 microplate reader. The intensity of lipid peroxidation was expressed as MDA content per unit of protein concentration.

### 2.8 GSH Assay

GSH levels were assessed using a GSH and GSSG assay kit (S0053, Beyotime). In brief, homogenized brain tissues (100 mg) or neurons (5 × 10^6^) were lysed in reagent M solution and then centrifuged. The supernatant was subsequently used to determine the total GSH content. The resulting solution was added to a 96‐well plate, mixed, incubated at 25°C for 5 min, and then mixed with 0.5 mg/mL NADPH. The absorbance was measured at 420 nm, and the data were normalized to unit protein concentration determined using a BCA kit.

### 2.9 Measurement of Cerebral Infarct Volume and Neurological Scores

To measure the cerebral infarction volume, the brains of mice were sliced into five 1‐mm‐thick coronal sections. The sections were next incubated in a 2% solution of 2,3,5‐triphenyl tetrazolium chloride (TTC, Sigma‐Aldrich, USA) at 37°C for 20 min in the dark and then photographed. The infarcted areas were calculated with ImageJ software by dividing the infarct volume by the total contralateral hemispheric volume. Neurological function in mice was measured using the Longa scores and the Neurological Severity Scores (NSS), as previously described [26, 27]. Behavioral tests were performed before pMCAO and at 24, 48, and 72 h after pMCAO by trained investigators who were blinded to the experimental design.

### 2.10 Statistical Analysis

The experimental data are presented as means ± standard deviation (SD). All data were analyzed and visualized using GraphPad Prism 7.0 software from at least three independent experiments. For all the data, we employed the Shapiro–Wilk test to assess their normality. Differences between the two groups were analyzed using the two‐independent‐sample *t*‐test. For the comparison of differences among multiple samples, one‐way ANOVA and least significant difference (LSD) were used for pairwise comparisons. For all data, differences with a *p*‐value of < 0.05 and a *p*‐value of < 0.01 were considered statistically significant, and those with a *p*‐value < 0.001 were considered highly statistically significant.

## 3 Results

### 3.1 Differentially Expressed mRNAs in OGD‐Treated Neurons Were Identified by RNA Sequencing

**FIGURE 1:**
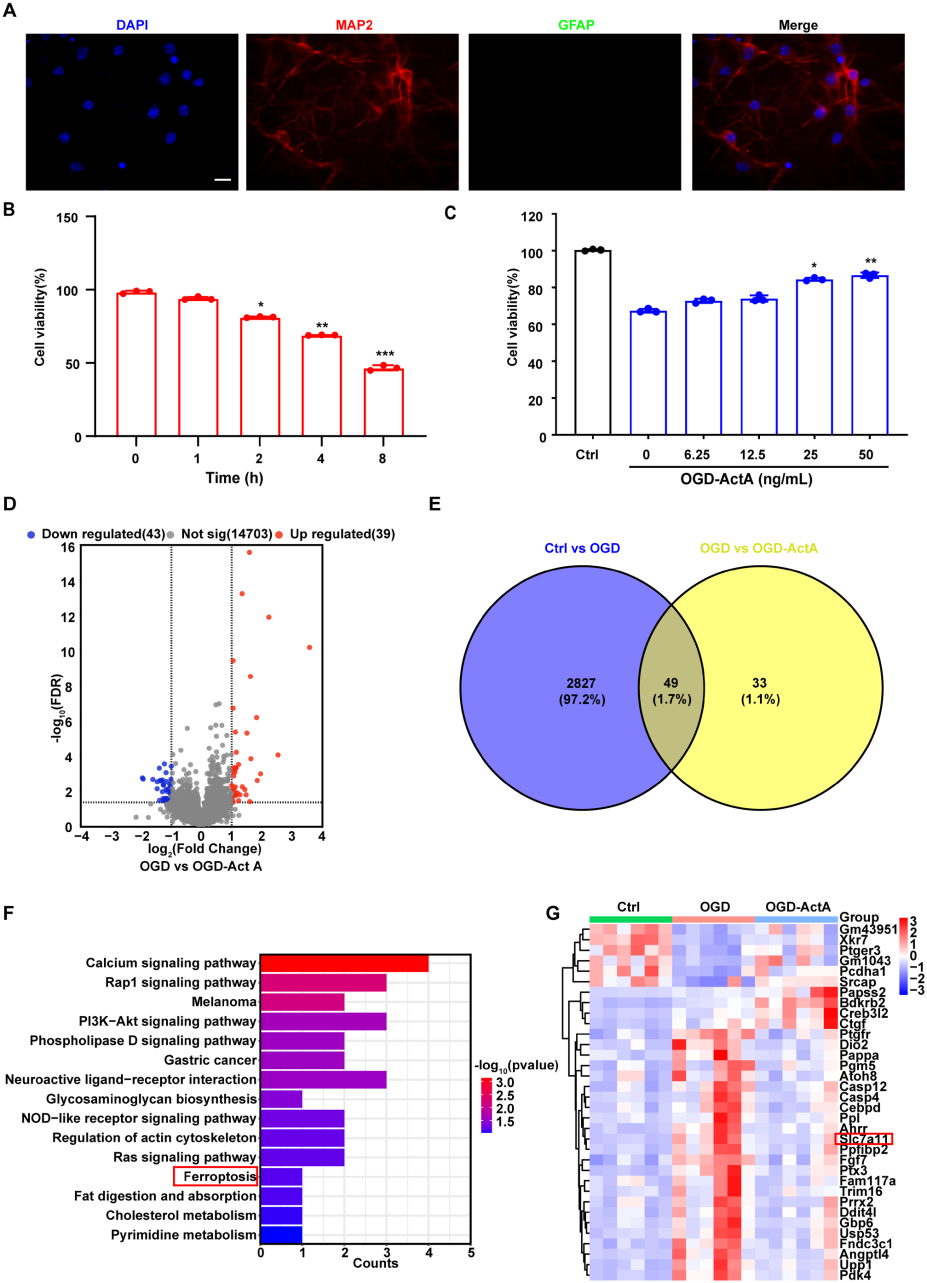
Identification of differentially expressed genes (DEGs) based on transcriptomics. (A) Immunofluorescent staining was performed using the neuronal marker microtubule‐associated protein 2 (MAP2) (red) and the astrocyte marker glial fibrillary acidic protein (GFAP) (green) antibodies to assess neurons. Nuclei were stained with DAPI (blue) (scale bar: 20 μm). (B) Time‐dependent changes in the viability of neurons exposed to oxygen–glucose deprivation (OGD) were detected by CCK‐8 assay. (C) Before 4 h of OGD neurons were pretreated with different concentrations of Act A for 30 min. (D) A volcano plot of the DEGs between the OGD and OGD‐Act A groups based on transcriptome sequencing results. Blue dots represent downregulated genes and red dots indicate upregulated genes. (E) A Venn diagram showing the 49 overlapping DEGs between the Ctrl versus OGD and the OGD versus OGD‐Act A transcriptomes. (F) KEGG pathway enrichment analysis of the 49 DEGs. The *x*‐axis represents the counts of DEGs involved in the pathway. (G) A heat map showing the expression of the 49 DEGs regulated by Act A after OGD. The colored column represents the sample groups and the row name indicates the DEGs; red denotes high expression and blue denotes low expression. The data are presented as means ± SD (*n* = 3; *p < 0.05, **p < 0.01, ***p < 0.001 compared with the Ctrl group or the OGD group).

Identification of cultured surviving cells as neurons by double staining of neuronal marker MAP2 and glial marker glial fibrillary acidic protein (GFAP) (Figure 1A). Primary cultured neurons were used to determine the neuroprotective effect of Act A after ischemic injury. As shown in Figure 1B, primary cultured neurons exhibited time‐dependent inhibition of cell viability following OGD. Subsequently, the impact of different concentrations of Act A on neurons exposed to OGD was examined. The results showed that Act A enhanced OGD‐treated neuron viability, indicating that Act A protected neurons against OGD‐induced damage. Furthermore, no significant difference in cell viability was observed between the 50 and 25 ng/mL Act A treatment groups (Figure 1C). Thus, 25 ng/mL Act A was used in subsequent experiments.

We further performed RNA‐sequencing analysis on primary neurons to reveal the effect of Act A treatment. We found that the administration of Act A notably downregulated the expression of 43 genes and upregulated that of 39 genes relative to OGD treatment alone (Figure 1D). To clarify the correlation between OGD‐Act A and OGD treatments, we compared the differentially expressed genes between the Ctrl and OGD groups with those differentially expressed between the OGD and OGD‐Act A groups (|log_2_FC| > 1), leading to the identification of 49 genes that were differentially expressed in both comparison groups (Figure 1E). The KEGG pathway analysis of the differentially expressed genes (DEGs) was conducted, which revealed the involvement of multiple signaling pathways, including the calcium signaling pathway, Rap1 signaling pathway, PI3K‐Akt signaling pathway, neuroactive ligand‐receptor interaction, regulation of actin cytoskeleton, Ras signaling pathway, and ferroptosis, as shown in Figure 1F. Both prior studies and our preliminary data demonstrate that multiple modalities of cell death—including apoptosis, autophagy, and ferroptosis—occur following cerebral ischemia [18, 28, 29]. However, the regulatory role of activin A on ferroptosis remains poorly characterized. Guided by RNA‐sequencing results, we therefore focused on elucidating the mechanism underlying activin A‐mediated ferroptosis inhibition following cerebral ischemia. Heatmap analysis indicated that Act A significantly downregulated the expression of a core marker of ferroptosis, *Slc7a11*, which encodes a cystine/glutamate xCT transporter (Figure 1G). qPCR analysis confirmed that the mRNA levels of *Slc7a11, Pdk4, Angptl4*, and *Creb3l2* were in line with the bioinformatics results, achieving statistical significance (Figure S1).

### 3.2 Act A Protected Against OGD‐Induced Neuronal Ferroptosis

**FIGURE 2:**
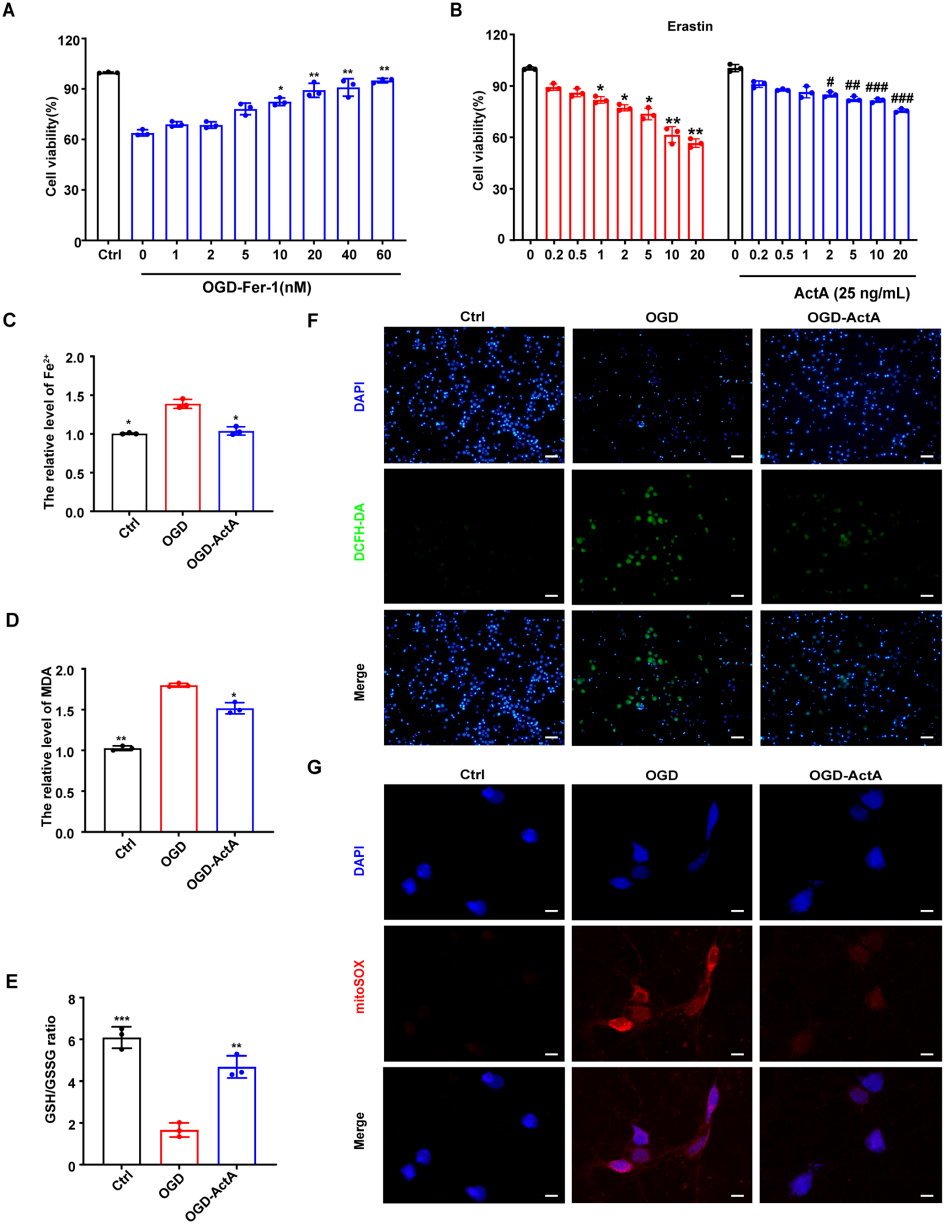
Inhibitory effects of Act A on neuronal ferroptosis induced by the oxygen–glucose deprivation (OGD). (A) The viability of OGD‐exposed primary cultured neurons treated with different doses of Fer‐1 was detected using the CCK‐8 assay. (B) The viability of primary cultured neurons stimulated by erastin for 24 h and treated with 25 ng/mL Act A for 24 h was examined using the CCK‐8 assay. (C) An iron assay was used to detect changes in intracellular Fe^2+^ levels in primary cultured neurons. (D) The extent of lipid peroxidation was measured using a malondialdehyde (MDA) assay kit. (E) GSH levels were measured using a GSH assay kit. (F) Representative images showed intracellular reactive oxygen species (ROS) levels (scale bar: 20 μm). (G) Representative images showed mitochondrial ROS levels in primary cultured neurons treated as indicated (scale bar: 20 μm). The data are presented as means ± SD (*n* = 3; *p < 0.05, **p < 0.01, ***p < 0.001 compared with the OGD group; ^#^ *p* < 0.05, ^##^ *p* < 0.01, ^###^ *p* < 0.001 compared with the erastin group).

Given the above results, we next sought to determine whether the protective effect of Act A was attributable to the inhibition of ferroptosis. Fer‐1, an effective inhibitor of ferroptosis, mitigated neuronal death in OGD‐induced injury, and Act A significantly protected against death induced by the ferroptosis inducer erastin (Figure 2A,B). These results implied that Act A reduced the occurrence of OGD‐induced neuronal ferroptosis.

**FIGURE 3:**
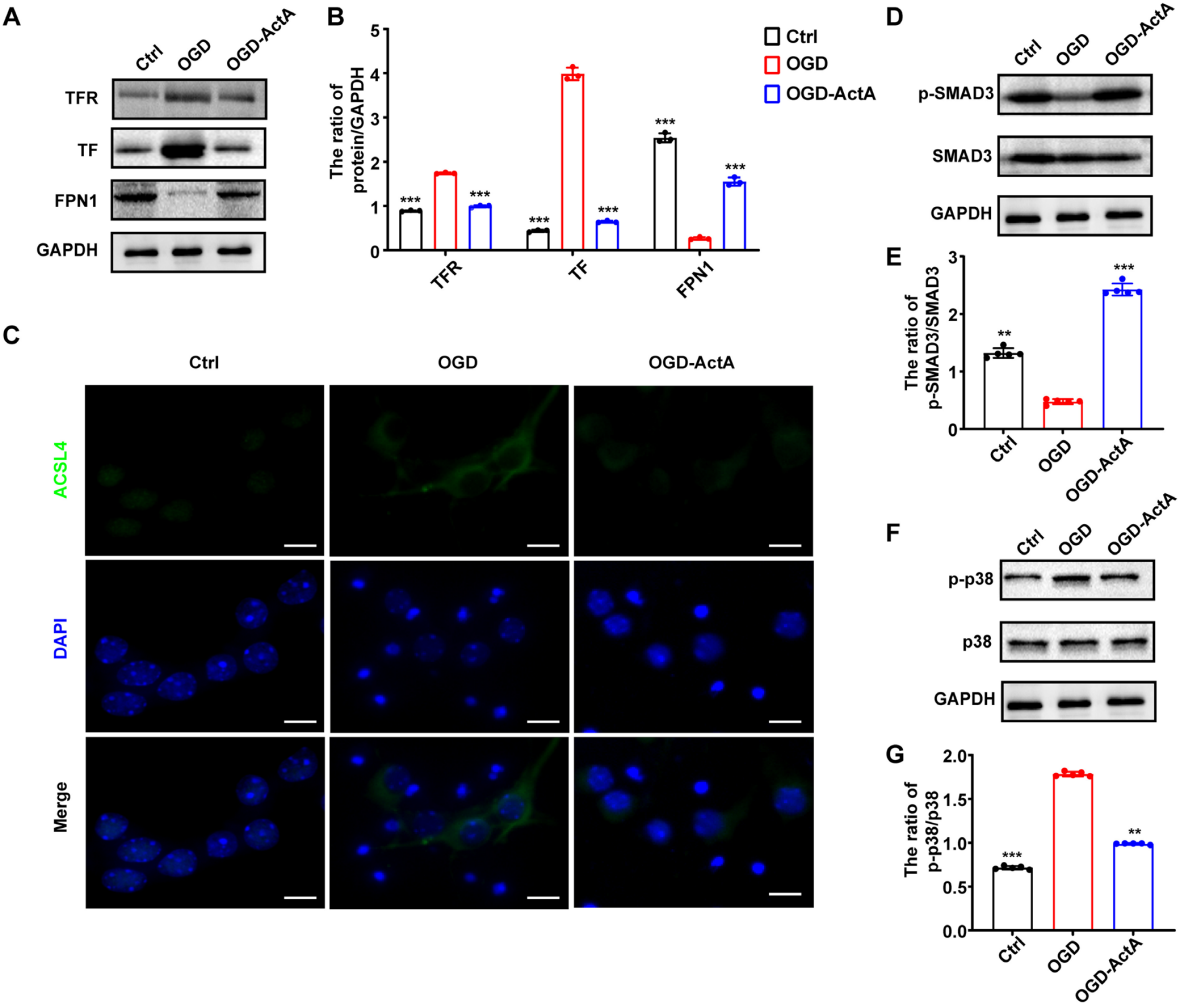
Expression of the ferroptosis‐related proteins and SMAD3 and p38 in neurons subjected to oxygen–glucose deprivation (OGD) treated by Act A. (A) Western blotting was performed to determine the expression of ferroptosis‐associated proteins in primary cultured neurons treated as indicated. (B) Quantification of the expression of TF, TFR, and FPN1 proteins. (C) Evaluation of ACSL4 (green) levels after OGD using immunofluorescence. Nuclei were stained with DAPI (blue). (Scale bar: 100 μm). (D, E) Western blotting was used to evaluate SMAD3 activation in primary cultured neurons treated as indicated. (F, G) Western blotting was performed to analyze the extent of p38 inhibition in primary cultured neurons treated as indicated. The data are presented as means ± SD (*n* = 3; *p < 0.05, **p < 0.01, ***p < 0.001 compared with the OGD group).

Additionally, intracellular Fe^2+^ levels in primary cultured neurons were detected. As expected, the Fe^2+^ levels in neurons were significantly elevated after OGD treatment; the effect was reversed by Act A pretreatment (Figure 2C). Elevated levels of MDA, the end product of lipid peroxidation, are correlated with enhanced oxidative injury [30]. Here, we found that Act A treatment markedly reduced MDA levels after OGD treatment (Figure 2D). The GSH/GSSG assay revealed that OGD markedly decreased the levels of GSH, but this effect was reversed by Act A pretreatment (Figure 2E). We also evaluated both intracellular and mitochondrial reactive oxygen species (ROS) production in OGD‐induced neurons. The results revealed that OGD resulted in the accumulation of intracellular and mitochondrial ROS, whereas pretreatment with Act A significantly counteracted these OGD‐induced increases (Figure 2F,G). Western blotting was employed to analyze the expression of ferroptosis‐associated proteins in OGD‐exposed neurons treated or not with Act A. The results showed that the protein expression of FPN1 was notably elevated in neurons subjected to OGD + Act A treatment in comparison to those exposed only to OGD, whereas the protein expression of TF and TFR was significantly decreased (Figure 3A,B). The expression of ACSL4 protein was further examined by immunofluorescence and was found to be significantly upregulated by OGD; however, pretreatment with Act A reversed this effect (Figure 3C). These results confirmed that Act A pretreatment attenuated OGD‐induced ferroptosis in neurons.

It has been reported that brain ischemia leads to the inhibition of the Act A/SMAD3 pathway and the activation of the p38 MAPK pathway, both of which perform essential roles in neuronal apoptosis following ischemic injury [16, 25]. To explore the role played by SMAD3 and p38 MAPK signaling in ferroptosis, we examined the phosphorylation levels of SMAD3 and p38 MAPK. Here, our data showed that OGD treatment markedly increased p‐SMAD3 levels and the ratio of p‐SMAD3/SMAD3, and increased p‐p38 MAPK levels and the ratio of p‐p38MAPK/p38 MAPK in primary cultured neurons; however, these OGD‐induced effects were reversed by Act A pretreatment (Figure 3D–G).

### 3.3 *Arip1* Heterozygous Knockout in Primary Cultured Neurons Attenuated OGD‐Induced Ferroptosis via the SLC7A11/GPX4 Pathway

**FIGURE 4:**
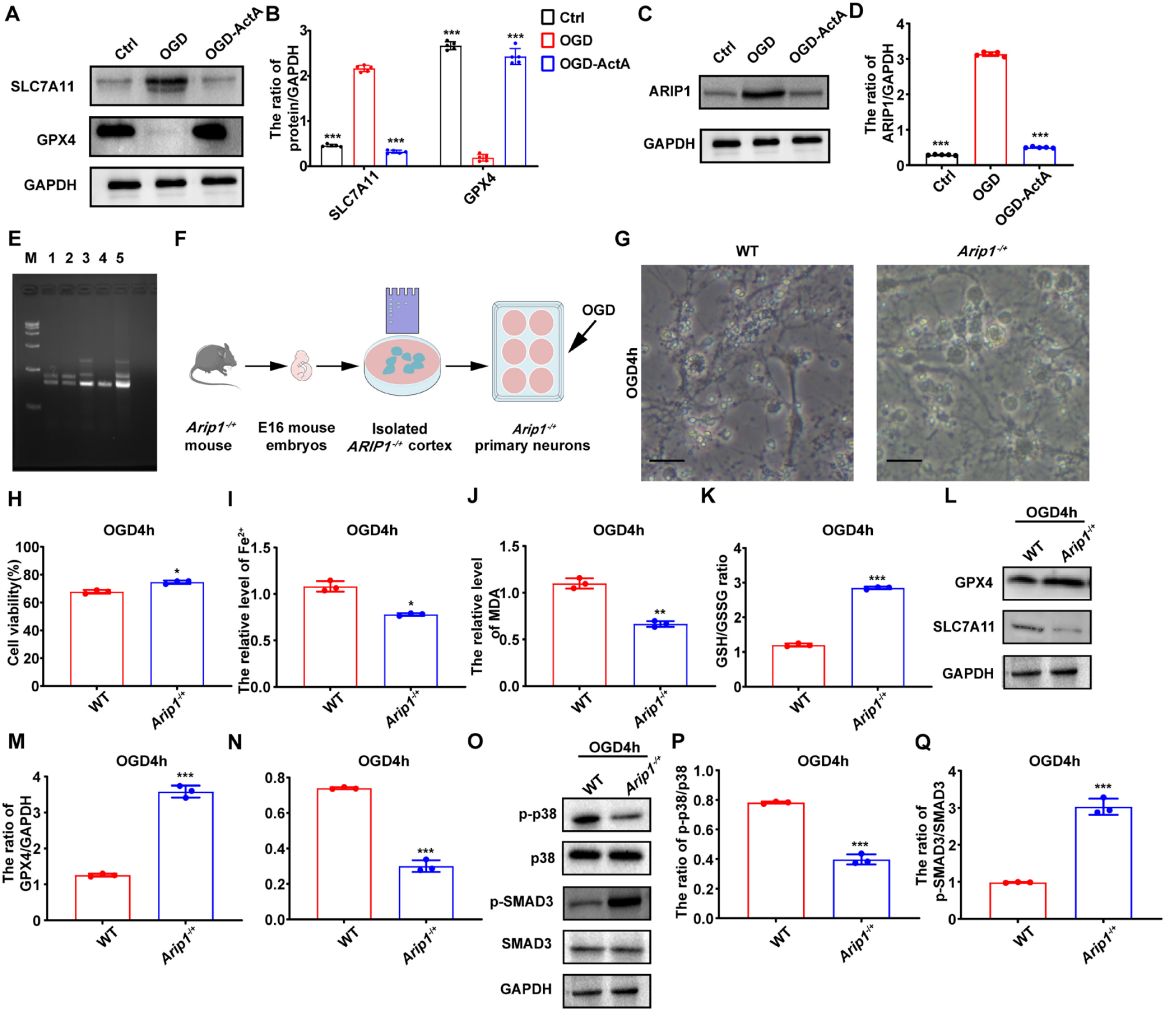
ARIP1 heterozygous knockout in primary neurons attenuated OGD‐induced ferroptosis via SLC7A11/GPX4 pathway. (A) Western blotting was performed to determine the expression of SLC7A11 and GPX4 proteins in primary cultured neurons treated as indicated. (B) Quantification analysis for SLC7A11 and GPX4. (C, D) Western blotting was used to examine ARIP1 protein expression in primary cultured neurons treated as indicated. (E) PCR identification of *Arip1* ^*−/+*^mice. M: Marker; Lanes 1, 2, 3, and 5: *Arip1* ^*−/+*^ mice; lane 4: Wild‐type (WT) mice. (F) Schematic illustration of *Arip1* ^*−/+*^ primary neuron extraction. (G) The morphologies of WT and *Arip1* ^−/+^ neurons were analyzed by microscopy (scale bar: 20 μm). (H) The viability of oxygen–glucose deprivation (OGD)‐exposed primary cultured neurons was detected by CCK‐8 assay. (I) The Fe^2+^ levels in WT and *ARIP1* ^*−/+*^ neurons. (J) The malondialdehyde (MDA) level in WT and *Arip1* ^*−/+*^ neurons. (K) The GSH level in WT and *Arip1* ^*−/+*^ neurons. (L–N) Western blotting was used to analyze SLC7A11 and GPX4 protein expression in primary cultured WT and *Arip1* ^*−/+*^ neurons after 4 h of OGD. (O–Q) Western blotting was used to analyze the levels of p‐SMAD3 and p‐p38 in cultured WT and *Arip1* ^*−/+*^ primary neurons after 4 h of OGD. The data are presented as means ± SD (*n* = 3; *p < 0.05, **p < 0.01, ***p < 0.001 compared with the OGD group).

To further validate the specific mechanism of ferroptosis inhibition by activin A, based on the RNA‐sequencing results, we evaluated the protein expression of SLC7A11 and ferroptosis‐associated protein GPX4 by western blotting. We found that OGD treatment significantly downregulated the expression of GPX4 and upregulated the expression of SLC7A11, whereas pretreatment with Act A resulted in the opposite effect (Figure 4A,B). Given ARIP1's established role as a negative regulator of activin A signaling through its interactions with ActRII and SMAD3, to investigate the specific regulatory role of ARIP1 under OGD conditions, the protein expression of ARIP1 was detected. As shown in Figure 4C,D, ARIP1 protein expression was markedly increased when neurons were subjected to OGD, whereas the administration of Act A significantly attenuated this increase. To identify whether ARIP1 expression is related to the inhibitory effect of activin A on neuronal ferroptosis, the heterozygous *Arip1* ^*−/+*^ mice (genotyped by DNA PCR followed by agarose gel electrophoresis; see Materials and methods) were used in the study (Figure 4E). Subsequently, the neurons from these mice were cultured in vitro and subjected to OGD (Figure 4F), following which changes in cell morphology were assessed by bright field microscopy. Wild‐type (WT) neurons were wrinkled and poorly adherent, and numerous suspended dead cells could be observed in the culture medium, in comparison to *Arip1* ^*−/+*^ neurons (Figure 4G). CCK‐8 assay results showed that neuronal survival in the *Arip1* ^*−/+*^ group was significantly higher than that in the WT group (Figure 4H). Additionally, following OGD, the levels of Fe^2+^ and MDA in *Arip1* ^*−/+*^ neurons were noticeably lower than those in WT neurons, while that of GSH showed the opposite tendency (Figure 4I–K). Western blotting results demonstrated that the protein expression of SLC7A11 was downregulated whereas that of GPX4 was elevated in *Arip1* ^*−/+*^ neurons following OGD treatment, which was consistent with the changes in expression recorded with Act A pretreatment (Figure 4L–N). These data provided direct evidence that ARIP1 deficiency enhanced the inhibitory role of Act A pretreatment in OGD‐induced ferroptosis, which subsequently modulated the SLC7A11/GPX4 pathway. Additionally, compared to the WT group, the level of p‐SMAD3 was prominently elevated in the *Arip1* ^*−/+*^ group, whereas that of p‐p38 exhibited the opposite effect (Figure 4O–Q). Collectively, these findings suggest that the SMAD3 and p38 MAPK pathways are involved in Act A action by which ARIP1 inhibits ferroptosis.

### 3.4 Act A Inhibited Ferroptosis in OGD‐Treated Neurons by Regulating the p38/SLC7A11/GPX4 and SMAD3/SLC7A11/GPX4 Pathways

**FIGURE 5:**
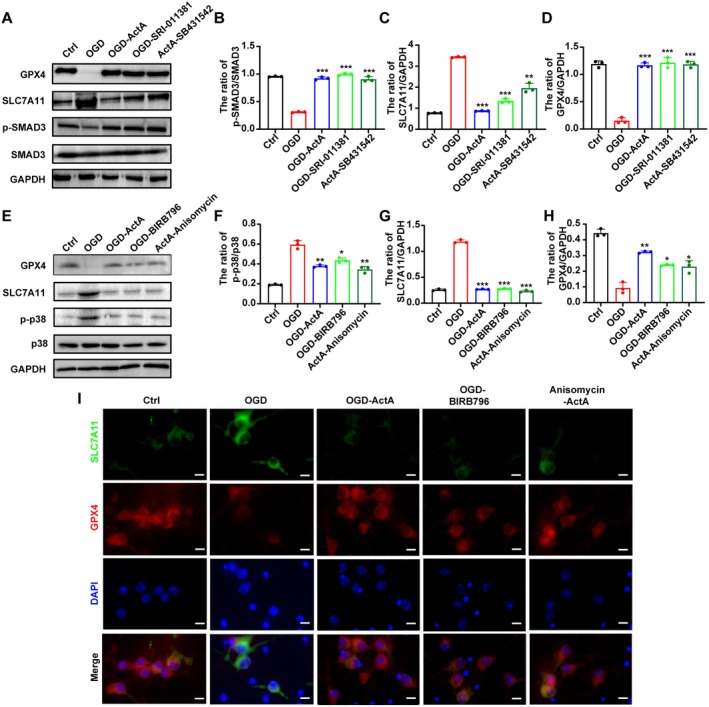
Expression of the SMAD3/SLC7A11/GPX4 and p38/SLC7A11/GPX4 in neurons treated by Act A. (A) Neurons were treated as indicated. Western blotting was performed to determine the protein levels of SLC7A11, GPX4, p‐p38, and p38. (B–D) Quantification analysis for the levels of p‐p38/p38, SLC7A11, and GPX4, respectively. (E) Western blotting analysis was performed to determine the protein levels of SLC7A11, GPX4, p‐SMAD3, and SMAD3 in primary cultured neurons treated as indicated. (F–H) Quantification analysis for the protein levels of p‐SMAD3/SMAD3, SLC7A11, and GPX4, respectively. (I) Neurons were treated as indicated, followed by immunofluorescence staining for SLC7A11 (green) and GPX4 (red). Nuclei were stained with DAPI (blue) (scale bar: 20 μm). The data are presented as means ± SD (*n* = 3; *p < 0.05, **p < 0.01, ***p < 0.001 compared with the OGD group).

To further confirm the potential mechanism underlying the roles of the SMAD3 and p38 MAPK pathways in OGD‐induced neuronal ferroptosis, we pretreated primary neurons with SRI‐011381, a specific SMAD agonist, or BIRB796, an inhibitor of p38, before OGD injury. As shown in Figure 5A–H, both SRI‐011381 and BIRB796 significantly inhibited the OGD‐induced increase of SLC7A11 protein and upregulated the expression of GPX4 protein. Subsequently, primary cultured neurons were treated with Act A in the presence of the SMAD inhibitor SB431542 or the p38 activator Anisomycin and undertook a western blotting analysis of the expression of SLC7A11 and GPX4. The results indicated that compared to the OGD group, SLC7A11 protein expression was markedly decreased in the Act A‐Anisomycin and Act A‐SB431542 groups. However, the protein expression of GPX4 showed the opposite tendency (Figure 5A–H). These results were not significantly different from those obtained with Act A treatment. Moreover, treatment with Anisomycin or SB431542 did not change the aforementioned effects mediated by Act A. SLC7A11 and GPX4 protein expression was further assessed using immunofluorescence, with similar results being obtained (Figure 5I). This suggested that Act A rescued OGD‐induced ferroptosis by enhancing the SMAD3 and inhibiting p38 MAPK signaling pathways, respectively, which act upstream of the SLC7A11/GPX4 pathway.

### 3.5 Neuronal Ferroptosis Was Detected in pMCAO Model Mice

**FIGURE 6:**
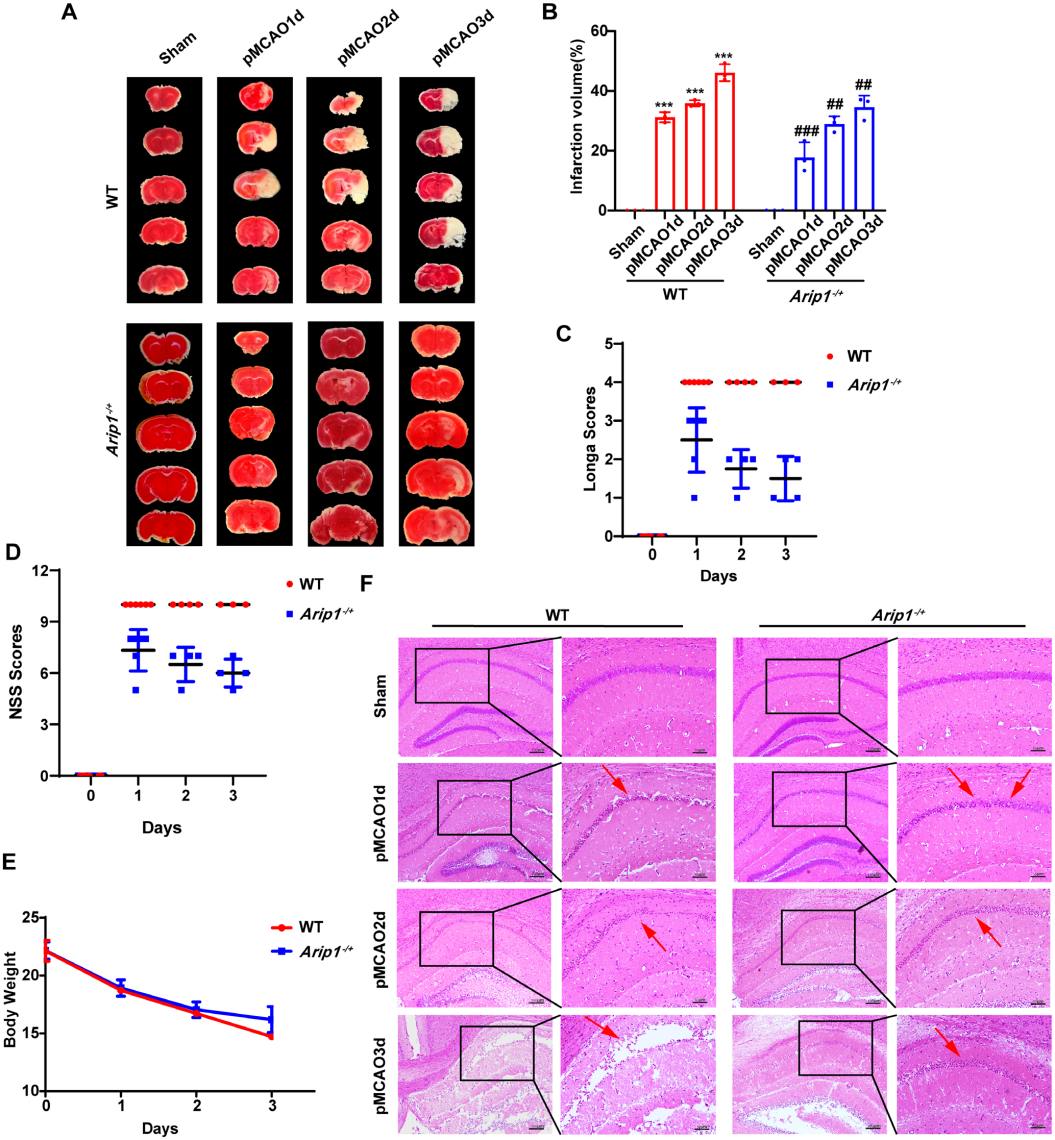
The heterozygous knockout of *Arip1* alleviated cerebral ischemic injury. (A, B) Cerebral infarction volume was evaluated by triphenyl tetrazolium chloride (TTC) staining. (C) Neurological deficits were assessed using the Longa scores. (D) Neurological severity scores in permanent middle cerebral artery occlusion (pMCAO) model mice. (E) Changes in body weight after ischemia. (F) Hematoxylin and eosin (H&E)‐stained sections of the CA1 region of the hippocampus from pMCAO mice in WT and *Arip1* ^*−/+*^ groups (scale bar: 100 μm). Data are expressed as means ± SD. *p < 0.05, **p < 0.01, ***p < 0.001 compared with the WT Sham group; ^#^ *p* < 0.05, ^##^ *p* < 0.01, ^###^ *p* < 0.001 compared with the WT group.

To observe whether ferroptosis occurs following cerebral ischemic injury, we established a pMCAO model in WT and *Arip1* ^*−/+*^ mice to mimic ischemic injury in vivo. Compared with Sham‐operated mice, the infarct volume in pMCAO model mice was significantly increased in WT mice (Figure 6A,B). Notably, the Longa and NSS scores were considerably higher in the group than in the Sham group (Figure 6C,D). The body weight of mice in the pMCAO group was significantly lower than that in the Sham group (Figure 6E). Hematoxylin and eosin (H&E) staining revealed that cerebral ischemia resulted in the loss of neuronal cells and nuclear consolidation in the CA1 region (Figure 6F). The results showed that pMCAO model mice were successfully prepared.

**FIGURE 7:**
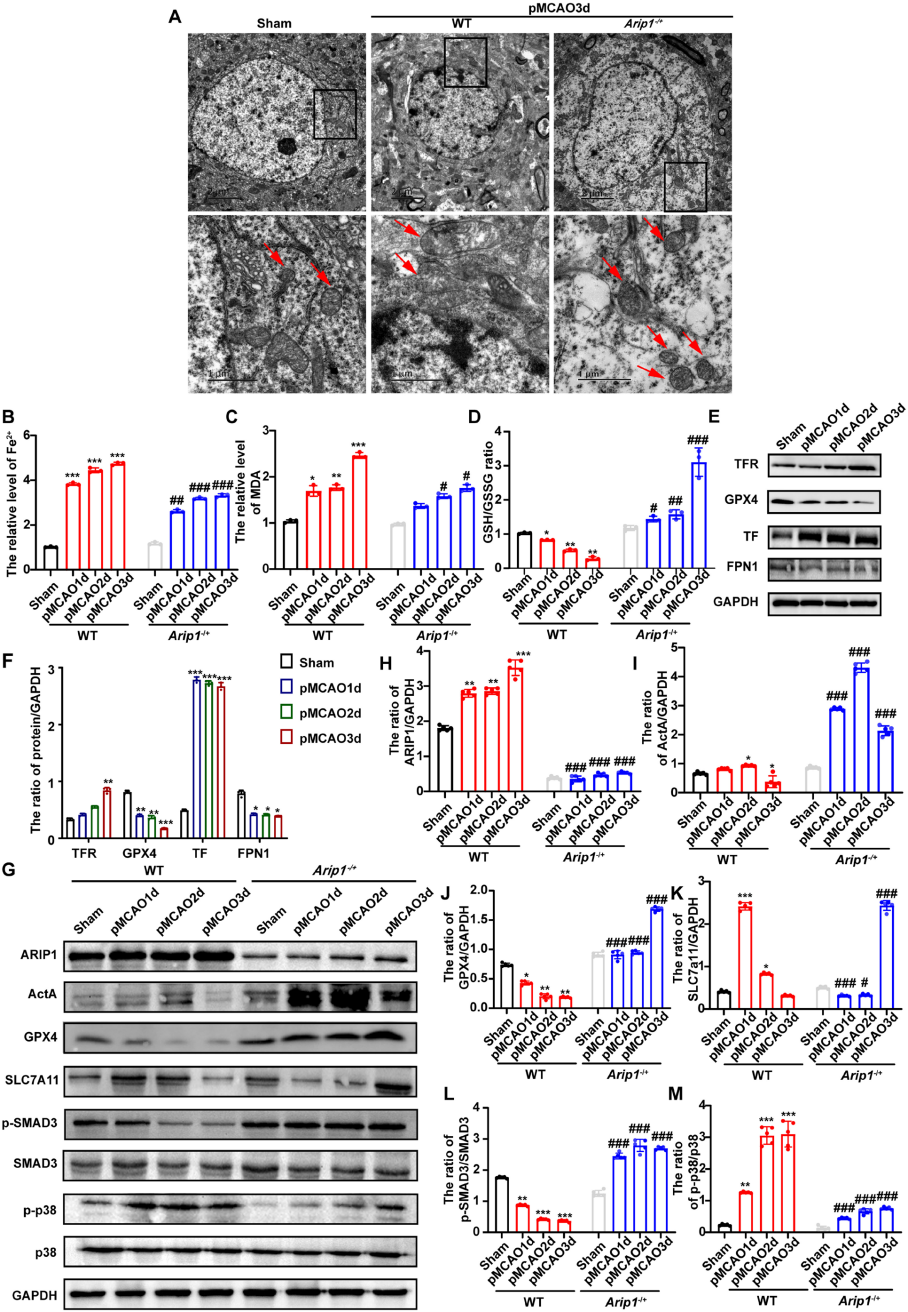
The SMAD3 and p38 MAPK pathways were involved and ferroptosis was inhibited in *Arip1* ^*−/+*^ mice after ischemia. (A) Transmission electron micrographs （TEM）of mitochondrial structures in cortical neurons of mice across different experimental groups (scale bar: 1 μm). (B) The Fe^2+^ levels in WT and *Arip1* ^*−/+*^ groups (*n* = 6 per group). (C) Malondialdehyde (MDA) levels in the WT and *Arip1* ^*−/+*^ groups (*n* = 6 per group). (D) GSH levels in the WT and *Arip1* ^*−/+*^ groups (*n* = 6 per group). (E) Western blotting was performed to determine protein levels in brain tissues of ischemic mice. (F) Quantification of the examined proteins. (G) Western blotting was performed to determine protein levels in the WT and *Arip1* ^*−/+*^ groups. (H–M) Quantification of the examined proteins. Data are expressed as means ± SD. *p < 0.05, **p < 0.01, ***p < 0.001 compared with the WT Sham group; ^#^ *p* < 0.05, ^##^ *p* < 0.01, ^###^ *p* < 0.001 compared with the WT group.

To elucidate the role of ferroptosis in the pathogenesis of ischemic injury, we examined the ultrastructural changes of mitochondria in ischemic brain tissue of pMCAO mice by transmission electron microscopy (TEM). Analysis of neurons in the cortex of mice on day 3 post‐pMCAO (pMCAO3d) showed numerous neurons containing swollen mitochondria, and fewer cristae were present compared with mitochondria in the Sham group (Figure 7A). As shown in Figure 7B, the Fe^2+^ and MDA contents in pMCAO model mice were significantly higher than those in Sham‐operated mice, whereas the GSH content was notably lower (Figure 7C,D). Subsequently, the levels of ferroptosis‐related proteins (GPX4, FPN1, TF, and TFR) were quantified by western blotting. We observed that after pMCAO, the protein expression of GPX4 and FPN1 was significantly reduced, whereas that of TF and TFR was greatly increased (Figure 7E,F). These findings demonstrated that cerebral ischemia injury affected the expression of ferroptosis‐related proteins, indicating that both iron transport and redox levels are affected by ischemia in pMCAO mice. Overall, our results suggested that ferroptosis is involved in the pathology of cerebral ischemia in mice.

To verify the changes in downstream signaling pathways following ferroptosis in cerebral ischemia, the expression of relevant proteins was examined. Compared with Sham‐operated mice, the protein levels of SMAD3, p38, p‐p38, ERK, p‐ERK, JNK, and p‐JNK were significantly elevated in the brain tissues of pMCAO model mice and gradually increased with time, while the levels of p‐SMAD3 exhibited the opposite trend (Figures S2 and 7G). These results suggested that ferroptosis during cerebral ischemia may be related to the SMAD and MAPK signaling pathways.

### 3.6 *Arip1* Heterozygous Knockout Alleviated Ferroptosis in Neurons by Regulating the SMAD3 and MAPK Pathways

The above in vitro findings suggest that ARIP1 regulates the SMAD3 and p38 MAPK signaling pathways, thereby inhibiting neuronal ferroptosis via the SLC7A11/GPX4 pathway following OGD injury. Hence, we next subjected *Arip1* ^*−/+*^ mice to pMCAO to validate the role of ARIP1 as a regulator of activin signaling in ferroptosis. As shown in Figure 6A,B, the pMCAO‐induced infarct volume was significantly reduced in *Arip1* ^*−/+*^ mice relative to that in their WT siblings. Following ischemia, *Arip1* ^*−/+*^ mice showed a significant decrease in the Longa and NSS scores, no significant change in body weight, a noteworthy increase in the number of surviving neurons in the CA1 region, and reduced nuclear consolidation, as determined by H&E staining, compared with WT mice. This indicates that *Arip1* ^*−/+*^ mice display a neuroprotective phenotype after ischemia (Figure 6C–F).

In Figure 7A, compared with the WT group, mitochondrial swelling was reduced and the number of cristae was significantly increased in *Arip1* ^*−/+*^ mice, as evidenced by TEM. In Figure 7B,C, after pMCAO, *Arip1* ^*−/+*^ mice showed a significant decrease in Fe^2+^ and MDA contents, and an increase in GSH levels relative to that observed in WT mice (Figure 7D). These results suggested that ferroptosis was significantly reduced in *Arip1* ^*−/+*^ mice after pMCAO. Next, the protein expression of Act A and the signaling proteins ARIP1, SMAD3, and p38 MAPK was examined by Western blotting (Figure 7G–I,L,M). With increasing ischemia duration, the protein expression of ARIP1 was upregulated in the WT group but not in the *Arip1* ^*−/+*^ group. The protein expression of Act A in the ischemic tissue of *Arip1* ^*−/+*^ mice was significantly higher than that in the ischemic tissue of WT mice. Additionally, both WT and *Arip1* ^*−/+*^ mice displayed a significant increase in Act A protein expression on days 1 and 2 following pMCAO, peaking on day 2 in both groups. These data suggested that the loss of one *Arip1* allele can lead to an increase in endogenous Act A levels. Furthermore, after cerebral ischemia, the level of p‐SMAD3 was significantly increased while that of p‐p38 was markedly decreased in *Arip1* ^*−/+*^ mice compared with those of WT rodents.

To further validate the mechanism involved in ferroptosis regulation in *Arip1* ^*−/+*^ mice, the expression of relevant proteins was examined. We found that after ischemia, the protein expression of GPX4 was significantly increased in *Arip1* ^*−/+*^ mice compared to that in WT mice (Figure 7G,J). In WT mice, SLC7A11 protein expression was significantly increased on days 1 and 2, subsequently decreasing on day 3. This implied that the upregulation of SLC7A11 may occur at the early stage of ischemic injury‐induced ferroptosis. Meanwhile, SLC7A11 expression in *Arip1* ^*−/+*^ mice exhibited the opposite tendency (Figure 7G,K). Taken together, these findings demonstrated that the *Arip1* deficiency attenuated neuronal ferroptosis after cerebral ischemia by increasing endogenous Act A levels, thereby modulating the Act A/SMAD3 and p38 MAPK signaling pathways, ultimately influencing the SLC7A11/GPX4 pathway.

## 4 Discussion

Cerebral ischemic injury causes neuronal death, and activin A is an important neuroprotective factor. Recently, ARIP1 has been mainly expressed in neurons and is involved in the regulation of activin‐specific signal transduction. We assessed the neuroprotective roles of Act A in neuronal ferroptosis following cerebral ischemia employing both in vivo and in vitro methods, with a heterozygous deficiency of ARIP1 (*Arip1* ^*−/+*^) mouse. Our data suggested that the deletion of the ARIP1 gene is conducive to the neuroprotective effect of activin A in inhibiting ferroptosis during cerebral ischemic injury.

Neuroprotection represents a traditional approach for antagonizing the cascade of injurious molecular events, including brain oxidative stress, neuroexcitotoxicity, and neuroinflammation that lead to irreversible cerebral ischemic injury [31, 32]. For decades, researchers have been investigating therapeutic options to alleviate neuronal cell death [33, 34, 35, 36, 37]. The early implementation of neuroprotective measures can mitigate the consequences of reperfusion injury and increase the number of patients who can benefit from treatment [38], highlighting the importance of neuroprotective therapies.

Act A, a member of the TGF‐β superfamily, is involved in multiple biological processes. Previous work showed that Act A administration notably increases the survival of striatal and hippocampal neurons after ischemic injury in perinatal rats and rescues hippocampal neurons in neonatal rats [39]. In addition, recent studies have shown that Act A is neuroprotective against ischemic stroke and can reduce post‐ischemic neuronal death by inhibiting apoptosis and autophagy. However, the specific molecular mechanisms by which Act A exerts its neuroprotective effects after cerebral ischemia remain unclear [16, 18]. Although all cells (including neurons, astrocytes, microglia, and endothelial cells) within the ischemic region are at risk for injury, neurons are likely the most vulnerable, and their death may be the most important factor contributing to clinical deficits in cerebral ischemia [40]. In the present study, we found that pretreatment with Act A significantly inhibited neuronal death due to OGD injury in primary cultured neurons. Additionally, RNA‐sequencing analysis indicated that Act A exerts its protective effects after cerebral ischemia via the regulation of OGD‐mediated ferroptosis.

Mounting evidence indicates that there is a close link between ferroptosis and oxidative stress, and the overproduction of ROS is thought to be the main cause of oxidative stress [41]. Similarly, excessive ROS production is a key pathological feature of the ischemic milieu [42, 43]. In this study, we detected intracellular and mitochondrial ROS levels in vitro using fluorescent probes, and found that Act A administration markedly mitigated the OGD‐induced increase in ROS production. The main biochemical features of ferroptosis include iron accumulation and lipid peroxidation [9]. Indeed, our results showed that Act A notably increases GSH levels while reducing iron levels, MDA production, and ROS accumulation. Meanwhile, RNA‐sequencing results indicated that the expression of the ferroptosis‐associated gene, *Slc7a11*, was significantly downregulated. *Slc7a11* encodes a cystine/glutamate antiporter that imports cystine into cells, supplying the substrate for GSH synthesis. However, under conditions of glucose deficiency or complete glutamine deficiency, the downregulation of SLC7A11 significantly boosts cell viability by enhancing intracellular glutamate utilization, thereby maintaining respiratory chain activity [44]. A recent study showed that xCT‐mediated extrasynaptic glutamate release is crucial for elevating extracellular glutamate concentrations following OGD [45]. It has also been demonstrated that during ischemic brain injury, HIF‐1α promotes xCT upregulation, leading to an increase in glutamate exocytosis, and, consequently, the activation of NMDAR, which can enhance neuronal iron uptake, suggestive of the onset of ferroptosis [13]. As expected, our findings demonstrated that the protein expression of SLC7A11 was elevated within 12 h in OGD‐treated primary cultured neurons but exhibited a significant decline after 24 h (Figure S3). Pretreatment with Act A abolished the changes induced after 4 h of OGD. Consistent with previous studies [46, 47], our data showed that the expression of GPX4 protein significantly decreased following OGD treatment; the effect was reversed by Act A pretreatment.

Recent studies revealed that both SMAD3 and p38 MAPK signaling are involved in cerebral ischemic injury, but the relationship between activin A and these signals is not clear in models of adult cerebral ischemia. ARIP1, abundantly present in the brain, has multiple protein–protein interaction domains that interact with both SMAD3 and ActRIIA. A previous study has reported that ARIP1 inhibits Act A signaling in neuronal cells. Meantime, our results confirmed that the expression of ARIP1 protein was upregulated after ischemia, both in vivo and in vitro, leading to pathological consequences such as oxidative stress, ferroptosis, and neurological deficits. Therefore, we speculate that ARIP1 is involved in the process by which activin A inhibits ferroptosis. Subsequently, we extracted primary neurons from *Arip1* ^*−/+*^ mice and found that *Arip1* heterozygous knockout inhibited ferroptosis and modulated the SLC7A11/GPX4 pathway through the activation of SMAD3 and the inhibition of the p38 MAPK signaling pathways, similar to that observed with Act A pretreatment. To identify the potential mechanisms underlying the effect of Act A treatment on ferroptosis, primary neurons were pretreated either with a specific p38 activator or an SMAD inhibitor. Our results showed that Act A suppressed the ferroptosis induced by a p38 agonist and a SMAD inhibitor. These findings demonstrated that Act A could reverse ischemic injury, offering notable evidence that activin A inhibits ferroptosis by regulating both SMAD3/SLC7A11/GPX4 and p38/SLC7A11/GPX4 pathways, which is facilitated by the deficiency of ARIP1.

In addition, several studies have demonstrated that Act A expression is elevated following cerebral injury at both the mRNA and protein levels [48, 49]. Indeed, our results revealed that Act A protein expression was rapidly upregulated in pMCAO model mice, peaking at postoperative day 2. However, the endogenous increases are not likely to be highly therapeutic but rather serve as biomarkers of injury. It has been shown that experimental doses of Act A are much greater than their endogenous levels after ischemia [25].

Thus, increasing endogenous Act A levels or activating activin A signaling may represent an effective therapeutic strategy for cerebral ischemia. As an in vivo model, we used *Arip1* ^*−/+*^ mice to validate the neuroprotective effects of endogenous Act A. Our results demonstrated that the heterozygous knockout of *Arip1* significantly promoted Act A and SMAD3 activities after ischemic injury. Moreover, we found that the deficiency of *Arip1* ^*−/+*^ exerted a neuroprotective effect on brain ischemia through the inhibition of ferroptosis, which activates the Act A/SMAD3 and inhibits p38 MAPK signaling pathways. The finding is consistent with the in vitro experiments. However, the mechanisms of these two signaling pathways in this process are not exactly the same. We will further explore this in depth later.

Interestingly, we found that SLC7A11 protein expression was upregulated after 3 days of ischemia, likely leading to an increase in intracellular cystine transport and enhanced GSH synthesis, ultimately inhibiting the occurrence of ferroptosis. We propose that xCT is engaged in two major biological processes following increased Act A protein expression after cerebral ischemia. Initially, xCT activity is reduced to suppress glutamate signaling, while over time, xCT activity increases to enhance redox signaling and oxidative stress protection, leading to the inhibition of ferroptosis. However, the molecular mechanism underlying this effect remains poorly understood. Recent work on transgenic mice expressing a dominant‐negative Act A receptor IB construct under the calmodulin kinase II‐alpha promoter has provided novel insights into the role of Act A [50]. Glutamatergic excitotoxicity is an essential component of cerebral ischemic injury; accordingly, the neuroprotective effects of Act A may result from a reduction in glutamatergic neurotransmission. However, in animals harboring a dominant‐negative Act A receptor, the blockade of Act A signaling results in an unexpected reduction in glutamatergic transmission. Further studies are needed to determine whether and how ARIP1 influences ferroptosis by altering glutamate signaling through SLC7A11 after ischemia. This will provide important insights into the mechanism by which Act A inhibits ferroptosis. In our study, we provided evidence that ARIP1 is a promising therapeutic target for ischemic brain damage.

This study has not yet fully expounded the relevant mechanisms. First, we only assessed the relationship between Act A and ferroptosis in cerebral ischemia, and other cell death pathways may also be involved in Act A's action. Studies have indicated that other forms of neuronal cell death, including apoptosis and autophagy, play important roles in the responses of ischemic neurons. Second, this study lacked in vivo studies on the dose and route of administration of Act A, which limits the clinical applicability of these findings. Future studies should address these limitations in light of the promising therapeutic potential of Act A in ischemic stroke.

## 5 Conclusions

In summary, in this study, we investigated the neuroprotective effects of Act A on cerebral ischemia‐induced ferroptosis and sought to identify the underlying mechanisms. Importantly, our study established that the neuroprotective properties of Act A involve the regulation of the SLC7A11/GPX4 pathway by ARIP1 via the modulation of the Act A/SMAD3 and p38 MAPK pathways. Furthermore, we verified that *Arip1* heterozygous knockout ameliorated neurological deficits and inhibited ferroptosis in a mouse model of pMCAO. *Arip1* ^*−/+*^ mice displayed increased levels of endogenous Act A, which subsequently influenced the SMAD3 and p38 MAPK pathways, leading to the inhibition of ferroptosis. These findings provide valuable insights into the pathogenesis of cerebral ischemia and the identification of potential therapeutic targets for the condition.

## Author Contributions

Z.Z.: conceptualization, methodology, formal analysis, writing – original draft, writing – review and editing, visualization. Y.Z.: methodology, validation, formal analysis, writing – review and editing. C.G.: validation, investigation, formal analysis. X.Q.: methodology, investigation. D.X.: validation. L.W.: data curation. Z.L.: writing – review and editing, supervision. H.L.: funding acquisition, project administration, writing – review and editing, supervision, resources.

## Disclosure

Lead Contact: Further information and requests for resources and regents should be directed to and will be fulfilled by the lead contact, Haiyan Liu (haiyan@jlu.edu.cn).

Materials Availability: This study did not generate new, unique reagents.

## Ethics Statement

The study was approved by the Experimental Animal Ethics Committee of Basic Medical College of Jilin University.

## Conflicts of Interest

The authors declare no conflicts of interest.

## Supporting information


**Table S1:** Primers used for qPCR.
**Figure S1:** mRNA levels were measured by qPCR in cultured primary neurons treated as indicated. The data are presented as means ± SD (*n* = 6; **p < 0.01, ***p < 0.001 compared with the OGD group).
**Figure S2:** Western blotting analysis of the level of p‐p38/p38, p‐ERK/ERK, and p‐JNK/JNK in pMCAO mice. Quantification of protein expression is presented in the right panel (*n* = 6, Data are expressed as mean ± SD. *p < 0.05, **p < 0.01, ***p < 0.001 compared with the Sham group.).
**Figure S3:** Western blotting analysis of SLC7A11 protein expression in primary neurons after OGD. Quantification of protein expression is presented in the right panel. The statistical data are represented as mean ± SD (*n* = 3; *p < 0.05, **p < 0.01, ***p < 0.001 compared with the Ctrl group).

## Data Availability

The data that support the findings of this study are available from the corresponding author upon reasonable request.
