# Supplementary material for: ARIP1 Deficiency Facilitates the Inhibition of Neuronal Ferroptosis in Cerebral Ischemia by Activin A Through SMAD3 and p38 MAPK Signaling
Source: CNS Neurosci Ther. 2025 Sep 18;31(9):e70615. doi: 10.1111/cns.70615 (PMC12445200; doi:10.1111/cns.70615)
Supplement: Supplementary file 1 — Table S1: Primers used for qPCR. Figure S1: mRNA levels were measured by qPCR in cultured primary neurons treated as indicated. The data are presented as means ± SD (n = 6; **p < 0.01, ***p < 0.001 compared with the OGD group). Figure S2: Western blotting analysis of the level of p‐p38/p38, p‐ERK/ERK, and p‐JNK/JNK in pMCAO mice. Quantification of protein expression is presented in the right panel (n = 6, Data are expressed as mean ± SD. *p < 0.05, **p < 0.01, ***p < 0.001 compared with the Sham group.). Figure S3: Western blotting analysis of SLC7A11 protein expression in primary neurons after OGD. Quantification of protein expression is presented in the right panel. The statistical data are represented as mean ± SD (n = 3; *p < 0.05, **p < 0.01, ***p < 0.001 compared with the Ctrl group). [file CNS-31-e70615-s001.docx]

**Supplementary Materials and Methods**

**Primary cortical neurons culture**

Primary mouse cortical neurons were obtained from E16 mouse embryos (C57BL/6J mice). Briefly, the dissected cerebral cortex was digested with trypsin. Free neurons were cultured in Neurobasal Medium (21103-049, Gibco) supplemented with 2% B27 (17504-044, Gibco) and 2 mM glutamine in a 5% CO2 incubator at 37 °C. Cultures were used for in vitro experiments after 10 days.

**CCK8 cell viability assay**

Primary neurons were seeded at a density of 1 × 104 cells per well in 96-well plates for 10 days and then treated accordingly (OGD or drug treatment, etc.). Next, 10 µL of CCK-8 solution was added in each well and incubated for 1 h using conventional culture conditions. The absorbance was recorded at 450 nm using a Tecan Austria GmbH reader (A-5082).

**ROS and MitoSOX fluorescence staining**

Intracellular reactive oxygen species (ROS) was measured using the 2′7′- dichlorodihydro-fluorescein diacetate (DCFH-DA) assay (Beyotime, China). The primary neurons were incubated with DCFH-DA in FBS-free DMEM for 15 min at 37 °C in the dark. Next, the cells were stained with Hoechst for 10 min at 37 °C. The level of ROS in the mitochondria was measured with the MitoSOX Red kit (M36008, Thermo Fisher). Neurons were incubated with 5 µM MitoSOX Red dissolved in FBS-free DMEM at 37 °C for 10 min and then washed three times with pre-warmed FBS-free DMEM. Nuclei were stained with DAPI (4′,6-diamidino-2-phenylindole) for 5 min. Pictures were subsequently taken on the ZEISS Fluorescent Microscope (Zeiss, Oberkochen, Germany). All the staining techniques and fluorescence observation were performed under light-avoidance conditions.

**Western blotting**

Collected brain tissues (ischemic middle cerebral artery regional cortex and corresponding regions of Sham-operated mice) and neurons were homogenized in RIPA buffer. Lysates were subjected to centrifugation at 12,000 × g, 15 min. The protein concentration was assessed using a BCA assay kit and boiled in loading buffer. Protein samples were separated on sodium dodecyl-sulfate polyacrylamide gels and transferred to polyvinylidene difluoride (PVDF) membranes. After blocking with 5% nonfat milk, the membranes were subsequently incubated with the following primary antibodies overnight at 4 °C: FPN1 (26601-1-AP, Proteintech), GPX4 (67763-1-Ig, Proteintech), TF (17435-1-AP, Proteintech), TFR (ab269513, Abcam), GAPDH (d110016, Sangon Biotech), Act A (26601-1-AP, Proteintech), SLC7A11 (ab216876, Abcam), p-SMAD3 (bs-5459R, Bioss), SMAD3 (abs1318272, Absin), p-p38 (9216S, Cell Signaling Technology), and p38 (8690S, Cell Signaling Technology). After TBS-T (Tris-buffered saline with 0.1% Tween 20) washing, the specific blots were incubated with the horseradish peroxidase-conjugated secondary antibodies for 1 h at room temperature. The secondary antibodies used in this study were as follows: goat anti-mouse (d110087, Sangon Biotech), goat anti-rabbit (d110058, Sangon Biotech) and goat anti donkey (SA00001-3, Proteintech). Finally, the protein bands were detected with an Amersham Imager 600 System (General Electric Company, Fairfield, CT, USA). Band patterns were analyzed with ImageJ software.

**Immunofluorescence staining**

The expression and localization of specific proteins in primary neurons were determined using immunofluorescence. Primary neurons were fixed with 4% paraformaldehyde for 30 min and blocked with 5% bovine serum albumin (BSA) containing 0.1% Triton X-100 for 20 min. Then they were incubated overnight at 4°C with primary antibody against GPX4 (67763-1-Ig, Proteintech), ACSL4 (ab227256, Abcam), and SLC7A1 (ab275411, Abcam). On the next day, the neurons were washed in PBS and then incubated for 1 h at room temperature with secondary antibodies conjugated with Alexa Fluor 488 (ab97050, Abcam) or Alexa Fluor 594 secondary antibody (M30010, Eugene, OR, USA) for 1 h at room temperature, and then DAPI (H-1200, Vector Laboratories) was stained for nucleus. We detected fluorescence with ZEISS Z2 Fluorescent Microscope (Zeiss, Oberkochen, Germany).

**pMCAO mice model**

Healthy male C57BL/6J mice (weighing 25 − 28 g, 8 − 10 weeks) were anesthetized by 1% sodium pentobarbital. A longitudinal midline incision in the neck was made, and common carotid artery (CCA), internal carotid artery (ICA), and external carotid artery (ECA) were isolated. Next, a 0.16 mm-diameter monofilament thread was inserted from the CCA to the ICA through a small incision in the CCA to the bifurcation of the middle cerebral artery. The middle cerebral artery was blocked for 24 h, 48 h, and 72 h. Sham-operated mice received the same surgical procedures without thread insertion.

**H&E staining**

The brains were perfused intracardially with PBS and fixed in 4% paraformaldehyde (PFA) at 4 ℃ for 24 h. After dehydration, they were paraffin-embedded and sectioned at 4 mm. Finally, the paraffin-embedded tissues were stained with hematoxylin and eosin (H&E). After sealing with resin, the histology of the cortex was observed by microscope (Olympus, Tokyo, Japan).

**Transmission electron microscopy of neurons**

Ischemic brain tissue was sliced and trimmed into 2 mm × 2 mm tissue blocks, which were subsequently fixed in 4% electron microscopy grade glutaraldehyde at 4°C for 24 h. Tissue blocks were rinsed in PBS and fixed in 1% osmium acid for 2 h. Afterward, the samples were dehydrated using graded concentrations of ethanol and acetone for 15 min and then immersed in Epon812 overnight. Finally, the sections were observed and recorded.

**Supplementary Table 1. Primers used for qPCR.**

| **Primer** | **Sequence (5′-3′)** |
| --- | --- |
| R-SLC7A11-F | CTCGTGACAGCTGTGGGCAT |
| R-SLC7A11-R | GGCACTAGACTCAAGAACTGTG |
| R-PDK4-F | TTTCCAGACCAACCAATTCACA |
| R-PDK4-R | TGCCCGCATTGCATTCTTA |
| R-ANGPTL4-F | CTCCCGTTAGCCCCTGAGAG |
| R-ANGPTL4-R | AGGTGCTGCTTCTCCAGGTG |
| R-CREB3L2-F | TGG TGG TTA CAA CCG CAG CA |
| R-CREB3L2-R | TCT TCT CCT CCT CTG TCA GGA C |
| R-GAPDH-F | TGCACCACCAACTGCTTAGC |
| R-GAPDH-R | GGCATGGACTGTGGTCATGAG |

**Supplementary figures and figure legends**


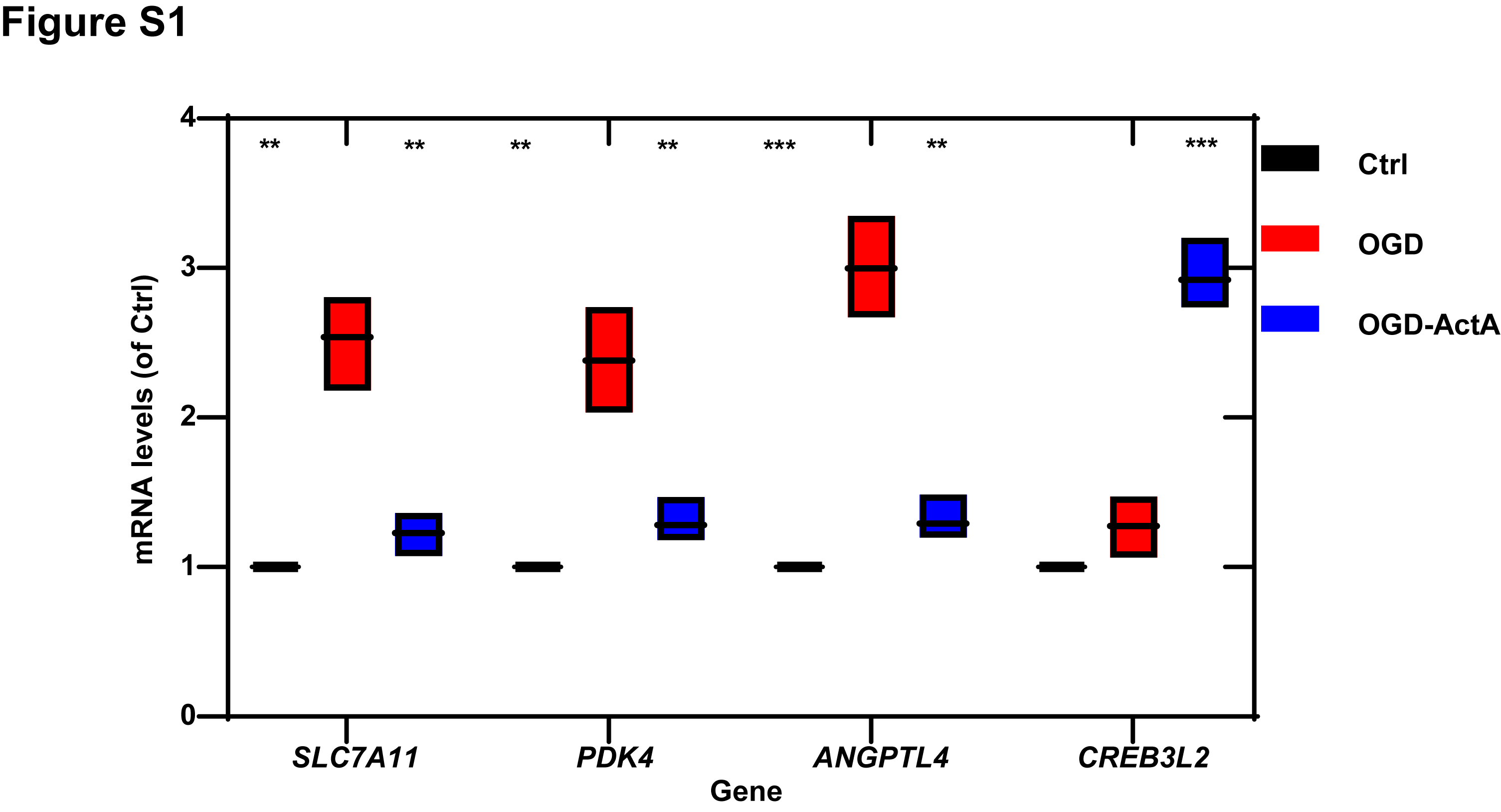


Figure S1 mRNA levels were measured by qPCR in cultured primary neurons treated as indicated. The data are presented as means ± SD (*n* = 6; ***P* < 0.01, ****P* < 0.001 compared with the OGD group).


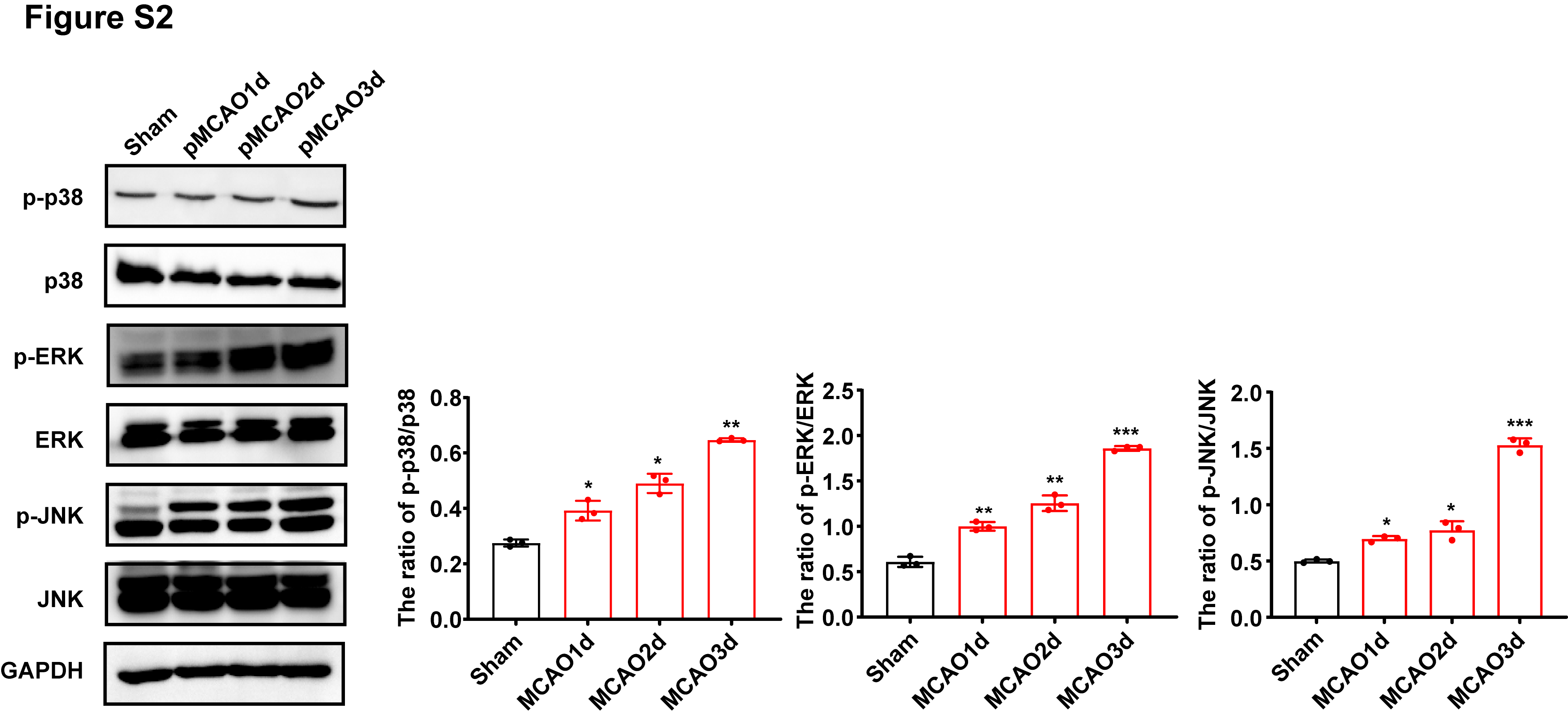


**Figure S2.** Western blotting analysis of the level of p-p38/p38, p-ERK/ERK, and p-JNK/JNK in pMCAO mice. Quantification of protein expression is presented in the right panel (n=6, Data are expressed as mean ± SD. **P* < 0.05, ***P* < 0.01, ****P* < 0.001 compared with the Sham group.).


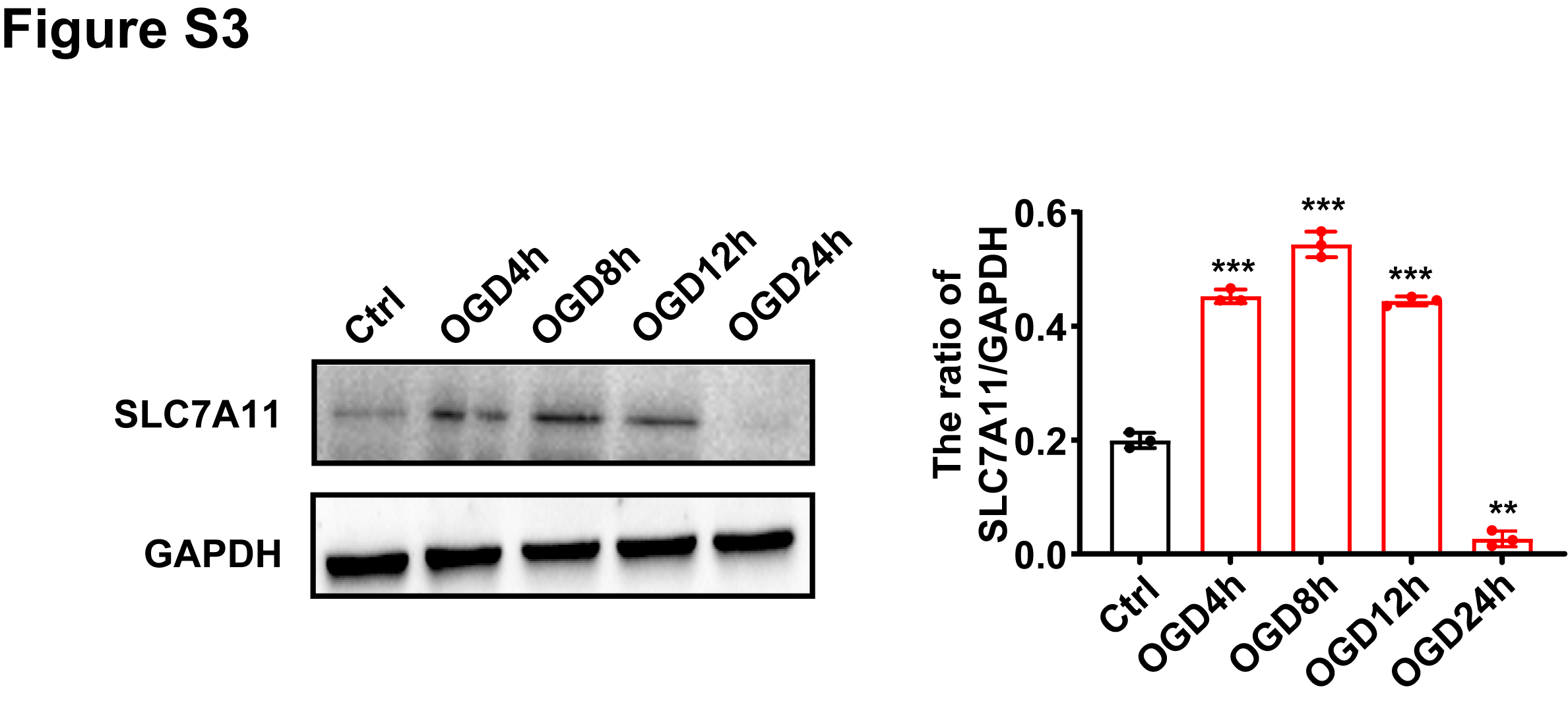


**Figure S3.** Western blotting analysis of SLC7A11 protein expression in primary neurons after OGD. Quantification of protein expression is presented in the right panel. The statistical data are represented as mean ± SD (n = 3; **P* < 0.05, ***P* < 0.01, ****P* < 0.001 compared with the Ctrl group).
